# A genome-wide CRISPR/Cas9 screen in acute myeloid leukemia cells identifies regulators of TAK-243 sensitivity

**DOI:** 10.1172/jci.insight.141518

**Published:** 2021-03-08

**Authors:** Samir H. Barghout, Ahmed Aman, Kazem Nouri, Zachary Blatman, Karen Arevalo, Geethu E. Thomas, Neil MacLean, Rose Hurren, Troy Ketela, Mehakpreet Saini, Moustafa Abohawya, Taira Kiyota, Rima Al-Awar, Aaron D. Schimmer

**Affiliations:** 1 Princess Margaret Cancer Centre, University Health Network, Toronto, Ontario, Canada.; 2 Department of Medical Biophysics, Faculty of Medicine, University of Toronto, Toronto, Ontario, Canada.; 3 Department of Pharmacology & Toxicology, Faculty of Pharmacy, Tanta University, Tanta, Egypt.; 4 Drug Discovery Program, Ontario Institute for Cancer Research, Toronto, Ontario, Canada.; 5 Leslie Dan Faculty of Pharmacy, University of Toronto, Toronto, Ontario, Canada.; 6 Institute of Medical Science, Faculty of Medicine, University of Toronto, Toronto, Ontario, Canada.; 7 Department of Biomedical Sciences, Zewail City of Science, Technology and Innovation, Giza, Egypt.; 8 Department of Pharmacology & Toxicology, University of Toronto, Toronto, Ontario, Canada.

**Keywords:** Oncology, Therapeutics, Cancer, Drug screens, Ubiquitin-proteosome system

## Abstract

TAK-243 is a first-in-class inhibitor of ubiquitin-like modifier activating enzyme 1 that catalyzes ubiquitin activation, the first step in the ubiquitylation cascade. Based on its preclinical efficacy and tolerability, TAK-243 has been advanced to phase I clinical trials in advanced malignancies. Nonetheless, the determinants of TAK-243 sensitivity remain largely unknown. Here, we conducted a genome-wide CRISPR/Cas9 knockout screen in acute myeloid leukemia (AML) cells in the presence of TAK-243 to identify genes essential for TAK-243 action. We identified BEN domain-containing protein 3 (*BEND3*), a transcriptional repressor and a regulator of chromatin organization, as the top gene whose knockout confers resistance to TAK-243 in vitro and in vivo. Knockout of *BEND3* dampened TAK-243 effects on ubiquitylation, proteotoxic stress, and DNA damage response. *BEND3* knockout upregulated the ATP-binding cassette efflux transporter breast cancer resistance protein (BCRP; ABCG2) and reduced the intracellular levelsof TAK-243. TAK-243 sensitivity correlated with BCRP expression in cancer cell lines of different origins. Moreover, chemical inhibition and genetic knockdown of BCRP sensitized intrinsically resistant high-BCRP cells to TAK-243. Thus, our data demonstrate that BEND3 regulates the expression of BCRP for which TAK-243 is a substrate. Moreover, BCRP expression could serve as a predictor of TAK-243 sensitivity.

## Graphical abstract

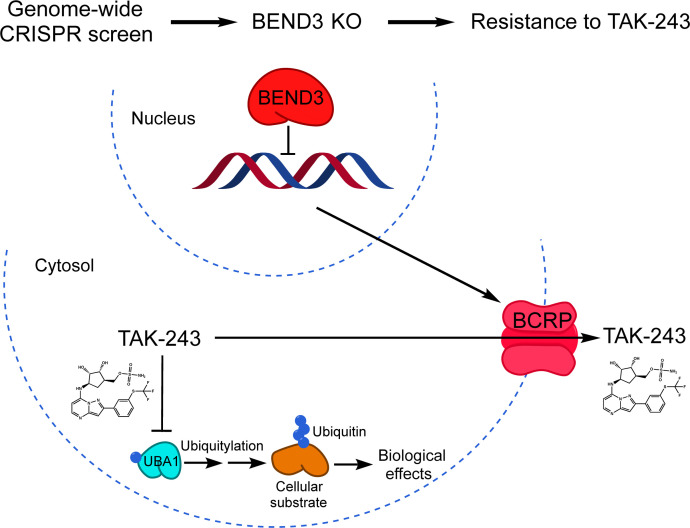

## Introduction

TAK-243 (also known as MLN7243) is a first-in-class inhibitor of the ubiquitin-like modifier activating enzyme 1 (UBA1) that catalyzes the first step of the ubiquitin conjugation cascade (1–3). Through this cascade, protein substrates are tagged with mono- or poly-ubiquitin to induce their proteasomal degradation or to modulate their functions (4, 5). This process is executed through multistep enzymatic reactions whereby ubiquitin is initially activated by the ubiquitin-activating enzyme (E1) in an ATP-dependent manner. This step is followed by the transfer of the activated ubiquitin from the catalytic cysteine site of E1 to the corresponding catalytic cysteine in one of the cognate ubiquitin-conjugating E2 enzymes (E2s). Ubiquitin is then transferred to protein substrates by E2s, and this step is facilitated by ubiquitin ligases (E3s). While UBA1 is the major ubiquitin E1 in the cell, there are over 30 ubiquitin E2s and hundreds of ubiquitin E3s that mediate the ubiquitylation of substrates in a highly coordinated and specific manner (6).

We previously reported that acute myeloid leukemia (AML) cell lines and primary patient samples are more dependent on the activity of UBA1 compared with normal hematopoietic cells and thus are more vulnerable to UBA1 inhibition (7). UBA1 was also reported by others to serve as a therapeutic target in cancer (8). Accordingly, we evaluated the selective UBA1 inhibitor, TAK-243, in preclinical models of AML and found that it displayed potent antileukemic activity in vitro and in vivo (9, 10). Similar findings have also been reported with TAK-243 in solid tumors and other hematologic malignancies (2, 11–13). Nonetheless, the determinants of sensitivity to TAK-243 are still largely unknown.

To gain further insights into the mechanisms of sensitivity and resistance to TAK-243, we conducted a genome-wide CRISPR/Cas9 knockout screen in AML cells and identified the transcriptional repressor BEN domain-containing protein 3 (*BEND3*) as the top gene whose knockout confers resistance to TAK-243.

## Results

### A genome-wide CRISPR/Cas9 knockout screen identifies BEND3 as a regulator of TAK-243 sensitivity.

TAK-243 is a first-in-class inhibitor of UBA1 that has been advanced to clinical trials (2, 10). To identify genes that influence the cytotoxicity of TAK-243, we performed a genome-wide CRISPR/Cas9 knockout screen in AML cells. OCI-AML2-Cas9 cells were transduced with a library of 91,320 guide RNAs (gRNAs) in lentiviral vectors targeting 17,232 genes at a ratio of 6 gRNAs per gene (14). Three days after transduction, cells were treated with TAK-243 at concentrations of the drug corresponding to the IC_90_ and IC_99_. Thirty-two days after the addition of TAK-243, surviving cells were harvested and the gRNA bar codes identified by sequencing. We focused our analysis on genes whose knockout conferred resistance to TAK-243.

**Figure 1:**
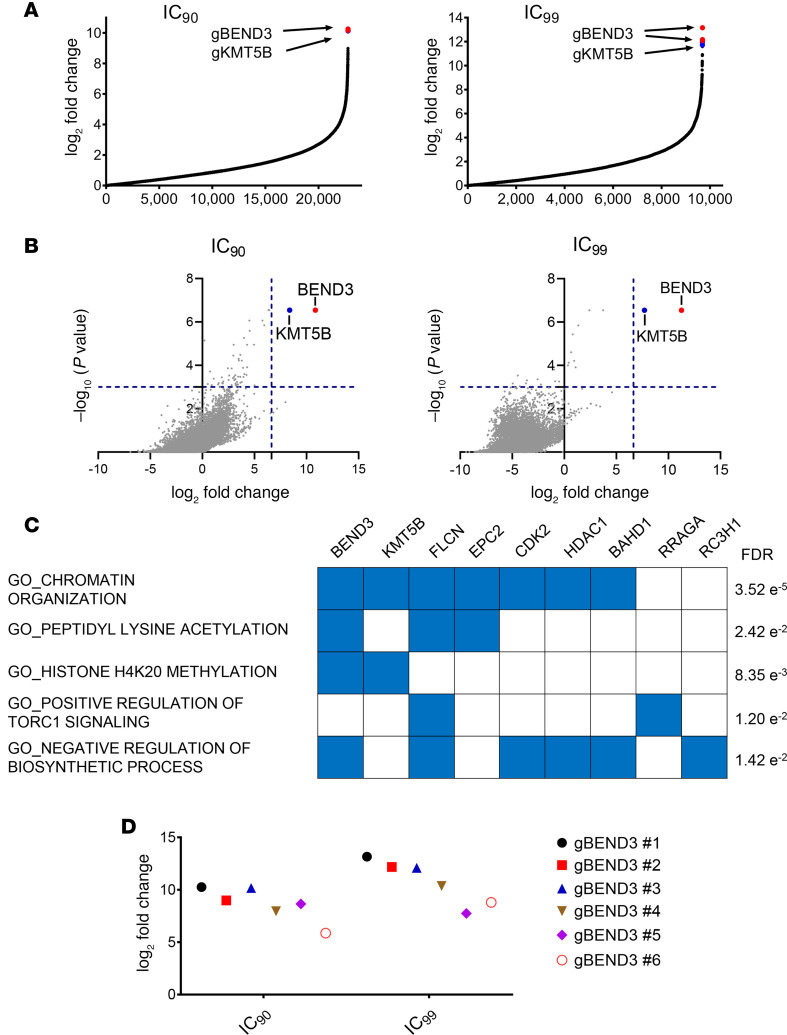
A genome-wide CRISPR/Cas9 knockout screen identifies *BEND3* as a top hit. (**A**) Enrichment of gRNAs after treatment with concentrations corresponding to the IC_90_ (left) and IC_99_ (right) of TAK-243 as assessed by fold change analysis compared with untreated control cells. Each data point represents a distinct gRNA. The gRNAs corresponding to *BEND3* and lysine methyltransferase 5B (*KMT5B*) are shown in red and blue, respectively. (**B**) Volcano plots representing log_2_ fold change of top enriched genes as ranked by the MAGeCK algorithm versus the significance of enrichment expressed as –log_10_ *P* value in the IC_90_ (left) and IC_99_ (right) arms of the screen. Each data point represents a distinct gene. Blue dashed lines are drawn at 100-fold enrichment (*x* axis) and a *P* value of 0.001 (*y* axis). Only *BEND3* (red) and *KMT5B* (blue) showed significant enrichment beyond these cutoff levels. (**C**) Gene set enrichment analysis (GSEA) showing Gene Ontology (GO) processes for genes whose gRNAs were significantly enriched in the IC_90_ arm of the screen and their corresponding false discovery rate (FDR) values. GO terms are shown on the left and the corresponding genes on the top. (**D**) Enrichment of gRNAs targeting *BEND3* in the IC_90_ and IC_99_ arms of the screen.

Of the 90,000 gRNAs in the CRISPR library, approximately 11,500 and 5500 gRNAs were enriched at least 2-fold at the IC_90_ and IC_99_ concentrations, respectively (Figure 1A). Using the Model-based Analysis of Genome-wide CRISPR/Cas9 Knockout (MAGeCK) algorithm to rank enriched genes (15) and a false discovery rate (FDR) of less than 0.2, 33 and 11 genes were identified as enriched in the populations of cells treated with TAK-243 at its IC_90_ and IC_99_ arms, respectively (Supplemental Tables 1–4; supplemental material available online with this article; https://doi.org/10.1172/jci.insight.141518DS1). At both IC_90_ and IC_99_ concentrations, *BEND3* ranked as the top hit (FDR = 0.001238; Figure 1B). Gene set enrichment analysis (GSEA) demonstrated that significantly enriched gRNAs corresponded to genes involved in diverse biological processes, including chromatin organization, peptidyl lysine acetylation, histone methylation, TORC1 signaling, and regulation of biosynthetic processes (Figure 1C). All 6 gRNAs targeting *BEND3* were enriched up to 1222- and 9136-fold after selection with TAK-243 at the IC_90_ and IC_99_ concentrations, respectively (Figure 1D). Based on this analysis, we focused our investigation on the top hit BEND3.

### BEND3 knockout confers resistance to TAK-243 in AML cells.

**Figure 2:**
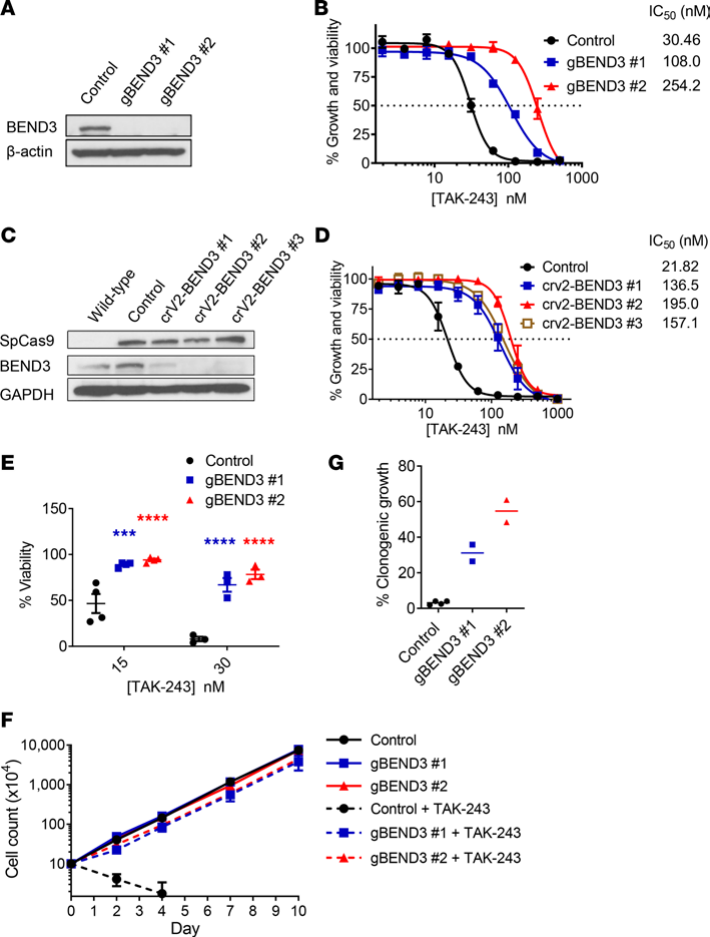
*BEND3* knockout confers resistance to TAK-243 in AML cells. (**A**) OCI-AML2 cells overexpressing Cas9 were stably transduced with gRNAs targeting *LacZ* (control) or BEND3. After transduction, whole cell lysates were prepared, and levels of BEND3 and β-actin serving as a loading control were measured by immunoblotting. (**B**) Control and *BEND3*-knockout OCI-AML2-Cas9 cells were treated with increasing concentrations of TAK-243 for 72 hours. Cell growth and viability was measured by the MTS assay. Inset: IC_50_ values (nM) are shown. Data points represent means ± SEM of 3 independent experiments. (**C**) WT OCI-AML2 cells were stably transduced with a single-plasmid system encoding spCas9 and gRNAs targeting *LacZ* (control) or *BEND3*. After transduction, whole cell lysates were prepared and levels of BEND3, spCas9, and GAPDH serving as a loading control were measured by immunoblotting. (**D**) Control and *BEND3*-knockout OCI-AML2-Cas9 cells were treated with increasing concentrations of TAK-243 for 72 hours. Cell growth and viability was measured by the MTS assay. Inset: IC_50_ values (nM) are shown. Data points represent means ± SEM of 3 independent experiments. (**E**) Control and *BEND3*-knockout OCI-AML2-Cas9 cells were treated with 2 concentrations of TAK-243 for 96 hours. Cell viability was measured by annexin V/PI staining and flow cytometry. Data points represent means ± SEM of 3–4 independent experiments. (**F**) Control and *BEND3*-knockout OCI-AML2-Cas9 cells were seeded with or without TAK-243 (30 nM), and trypan blue–negative cells were counted every 2–3 days. Data points represent means ± SEM of 2–3 counts. (**G**) Control and *BEND3*-knockout OCI-AML2-Cas9 cells were treated with TAK-243 (30 nM) and then plated into colony-forming assays. After 7 days of incubation, colonies of at least 50 cells were counted. The *y* axis shows the number of colonies as a percentage of the DMSO-treated controls taking into account plating efficiency as detailed in the Methods section. ***P ≤ 0.001; ****P ≤ 0.0001 using 2-way ANOVA and Sidak’s multiple comparisons test.

To validate the screen results, we knocked out *BEND3* using independent gRNAs. OCI-AML2-Cas9 cells were stably transduced with gRNAs targeting *BEND3* or control sequences, and knockout of *BEND3* was confirmed by immunoblotting (Figure 2, A and C). *BEND3*-knockout cells were then treated with increasing concentrations of TAK-243 and growth and viability measured by the MTS assay. *BEND3* knockout conferred resistance to TAK-243 with up to a 9-fold increase in the IC_50_ of the drug (Figure 2, B and D). *BEND3* knockout also conferred resistance to TAK-243 as measured by annexin V/propidium iodide (PI) staining and proliferation assays using trypan blue dye exclusion (Figure 2, E and F). Finally, knockout of *BEND3* reduced the ability of TAK-243 to target the colony-forming cells as measured by clonogenic assays (Figure 2G). Of note, *BEND3* knockout had little or no impact on cell proliferation rate in the absence of TAK-243 treatment (Figure 2F).

### BEND3 knockout confers resistance to TAK-243 in vivo.

**Figure 3:**
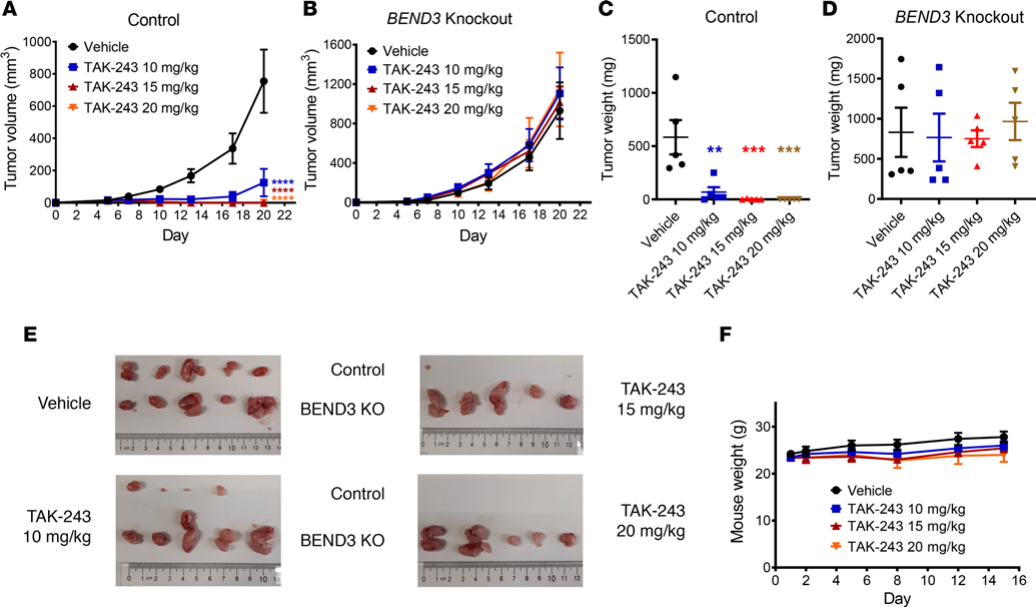
*BEND3*-knockout AML tumors are resistant to TAK-243 in a mouse xenograft model. (**A** and **B**) Control (**A**) and BEND3-knockout (**B**) OCI-AML2 cells (1 × 10^6^) were injected subcutaneously into the right and left flanks of SCID mice, respectively. When the tumors became palpable, mice were randomly divided into 4 groups (*n* = 5 per group) and treated with vehicle (10% 2-hydroxypropyl-β-cyclodextrin [HPBCD] in water) or TAK-243 (10, 15, and 20 mg/kg) subcutaneously twice weekly for 3 weeks. Asterisks shown denote significantly different final tumor volumes in TAK-243–treated groups compared with vehicle, determined using repeated-measure 2-way ANOVA and Sidak’s multiple comparisons test. (**C** and **D**) After 3 weeks, mice were euthanized and tumors of control (**C**) and *BEND3*-knockout (**D**) OCI-AML2 cells harvested and weighed. Significance of difference was determined using 1-way ANOVA and Tukey’s multiple comparisons test. (**E**) Images of control (top) and *BEND3*-knockout (bottom) OCI-AML2 tumors harvested from the 4 groups are shown. (**F**) Mice were weighed every 2–3 days. Data points in **A**–**D** and **F** represent means ± SEM of a representative experiment (*n* = 2). **P ≤ 0.01; ***P ≤ 0.001; ****P ≤ 0.0001.

Next, we determined whether BEND3 regulates the sensitivity of AML cells to TAK-243 in vivo. Control or *BEND3*-knockout OCI-AML2 cells were injected into severe combined immunodeficiency (SCID) mice. After the tumors became palpable, mice were treated with increasing doses of TAK-243 subcutaneously twice weekly (BIW). As previously described (10), TAK-243 produced dramatic reductions in tumor growth in WT OCI-AML2 cells (Figure 3, A, B, and E). In contrast, *BEND3* knockout rendered the tumors resistant to TAK243, and thus they grew at a rate similar to control (Figure 3, C–E). Of note, *BEND3*-knockout cells exhibited a tumor growth rate in vivo comparable to that of control cells in vehicle-treated mice, which is consistent with proliferation data observed in vitro (Figure 3, A, C, and E). All TAK-243 doses were tolerated as evidenced by nonsignificant changes in mice weights in TAK-243– versus vehicle-treated mice (Figure 3F).

### BEND3 knockout dampens TAK-243 effects on ubiquitylation, proteotoxic stress, and DNA damage response in AML cells.

**Figure 4:**
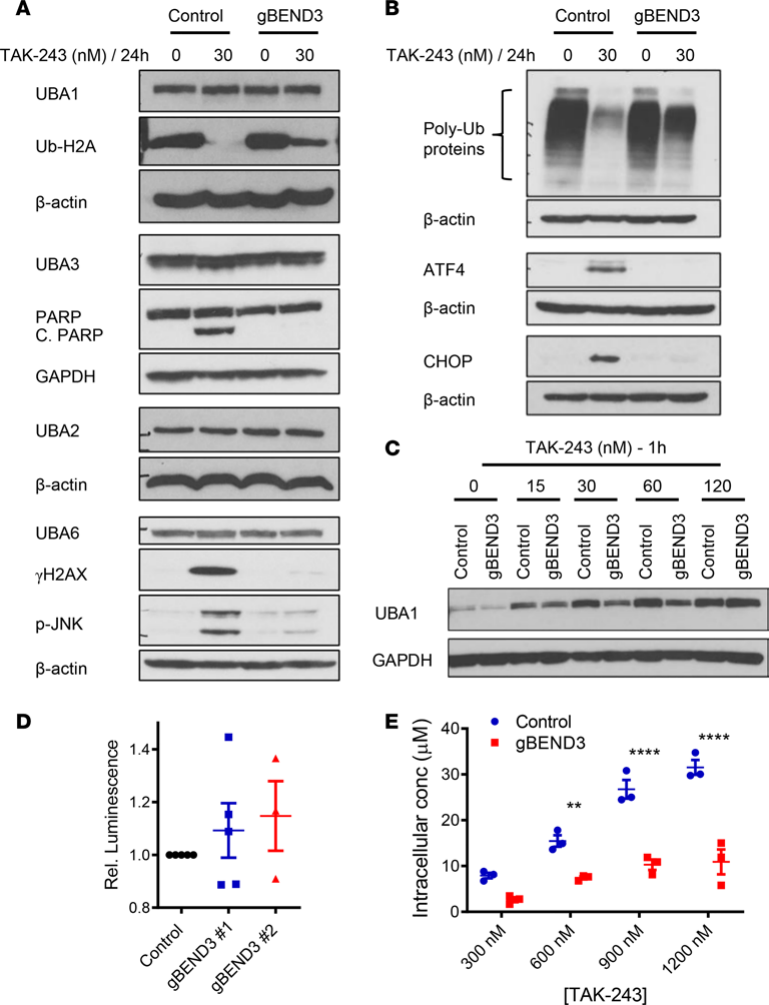
*BEND3* knockout dampens TAK-243 effects and reduces the intracellular transport of TAK-243 into AML cells. (**A** and **B**) Control and *BEND3*-knockout OCI-AML2-Cas9 cells were treated with DMSO or TAK-243 (30 nM) for 24 hours. After treatment, whole cell lysates were prepared, and levels of UBA1, UBA3, UBA6, UBA2, poly-ubiquitylated proteins, activating transcription factor 4 (ATF4), poly (ADP-ribose) polymerase (PARP), cleaved PARP (C. PARP), DNA-damage inducible transcript 3 (CHOP), phospho-JNK (p-JNK), and Ser^139^ phosphorylated H2AX (γH2AX) were measured by immunoblotting. GAPDH and β-actin were used as loading controls. (**C**) Control and *BEND3*-knockout OCI-AML2-Cas9 cells were treated with DMSO or increasing concentrations of TAK-243 at 15–120 nM for 1 hour followed by heating the intact cells at 54°C. After heating, whole cell lysates were prepared, and levels of UBA1 and GAPDH were measured by immunoblotting. (**D**) Control and *BEND3*-knockout OCI-AML2-Cas9 cells were washed, seeded in equal numbers, and lysed. Luminescence was then measured after adding an ATP-dependent luciferase reagent. Relative luminescence obtained from *BEND3*-knockout OCI-AML2-Cas9 cells was calculated by normalizing to control cells. Data points represent means ± SEM of 3–5 independent experiments. (**E**) Control and *BEND3*-knockout OCI-AML2 cells were treated with increasing concentrations of TAK-243 (300–1200 nM) for 1 hour and washed, and pellets were then extracted with acetonitrile. TAK-243 concentrations were then measured by LC-MS. Data points represent means ± SEM of triplicate data from a representative experiment (*n* = 2). **P ≤ 0.01; ****P ≤ 0.0001 using 2-way ANOVA and Sidak’s multiple comparisons test.

TAK-243 inhibits UBA1, leading to reductions in poly- and mono-ubiquitylation, with the resultant induction of proteotoxic and DNA damage stress and subsequent cell death (2, 10). To determine how BEND3 influences sensitivity to TAK-243, we treated control and *BEND3*-knockout OCI-AML2-Cas9 cells with TAK-243 and measured changes in the levels of UBA1, the abundance of ubiquitylated proteins, and markers of proteotoxic and DNA double-strand break repair. *BEND3* knockout did not change protein levels of UBA1 or other related E1s (Figure 4A). However, it attenuated TAK-243–induced reductions in both poly-ubiquitylation and H2A mono-ubiquitylation (Figure 4, A and B). In keeping with this finding, TAK-243–treated *BEND3*-knockout cells exhibited a little or no induction of markers of proteotoxic stress (ATF4, CHOP, and p-JNK), DNA damage (γH2AX), and apoptosis (PARP cleavage) (Figure 4, A and B).

### BEND3 knockout reduces the intracellular transport of TAK-243 into AML cells.

TAK-243 is an AMP mimetic that binds to the nucleotide-binding site of the UBA1 enzyme in an ATP-competitive manner and then forms a covalent adduct with ubiquitin in a reaction requiring UBA1 activity. The resulting TAK-243–ubiquitin adduct inhibits UBA1 (2). We used the cellular thermal shift assay (CETSA) to evaluate the binding of TAK-243 to UBA1 in control versus *BEND3*-knockout OCI-AML2-Cas9 cells. Control and *BEND3*-knockout cells were treated with increasing concentrations of TAK-243 followed by measuring the thermal shift of UBA1 by immunoblotting. As assessed by this assay, *BEND3* knockout reduced TAK-243 binding to UBA1 (Figure 4C). However, it did not change the intracellular levels of ATP, indicating that resistance to TAK-243 could not be explained by increased levels of ATP that competes for UBA1 binding (Figure 4D).

To assess the accumulation of TAK-243 into OCI-AML2-Cas9 cells, we measured intracellular TAK-243 concentrations following treatment with increasing concentrations of the drug for 1 hour. As assessed by liquid chromatography–mass spectrometry (LC-MS), knockout of *BEND3* reduced the intracellular concentrations of TAK-243 compared with control (Figure 4E).

### Upregulation of breast cancer resistance protein mediates TAK-243 resistance in vitro and in vivo.

The emergence of multidrug resistance (MDR) is a common problem with antineoplastic agents, including cytotoxic drugs and molecularly targeted therapeutics (16). A major class of proteins mediating MDR are the ATP-binding cassette (ABC) transporters that act as efflux pumps to extrude drugs and xenobiotics out of the cells in an ATP-dependent manner (17). Since *BEND3* knockout reduced the accumulation of TAK-243 into AML cells, we hypothesized that the upregulation of one or more ABC transporters may be responsible for the resistance phenotype.

**Figure 5:**
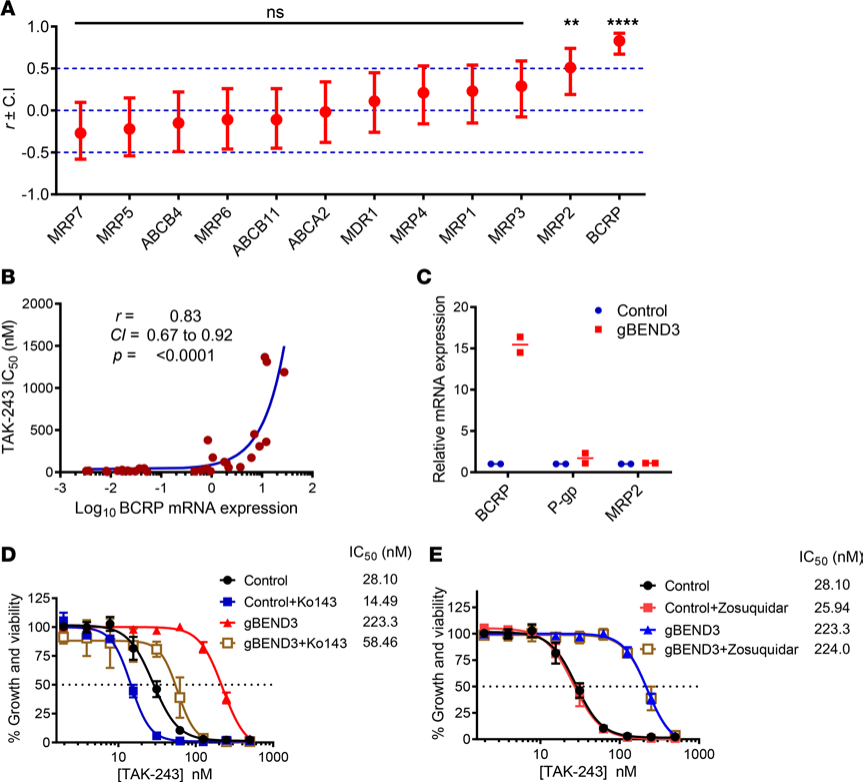
Upregulation of BCRP mediates TAK-243 resistance upon *BEND3* knockout in AML cells. (**A**) RNA-Seq expression data of 12 ABC transporters were obtained from the Cancer Cell Line Encyclopedia and correlated with TAK-243 sensitivity (as measured by IC_50_) of 30 cell lines. The *x* axis represents the ABC transporters, and the *y* axis represents the value of the linear Pearson correlation coefficient (*r*) ± upper and lower confidence intervals (CIs) for each transporter. The significance of correlation is shown on the graph. **P ≤ 0.01; ****P ≤ 0.0001. (**B**) Correlation curve of the mRNA expression of BCRP (ABCG2) and TAK-243 sensitivity (as measured by IC_50_). Data points represent the 30 cell lines used in the analysis. A logarithmic scale was used for the *x* axis to display all the data points over a wide range. Inset: the Pearson correlation coefficient (*r*), CI, and significance of correlation (as assessed by *P* value). (**C**) Relative mRNA expression of BCRP, P-gp, and MRP2 in control and *BEND3*-knockout OCI-AML2-Cas9 cells as assessed by RT-qPCR. Data points represent means of 2 biological replicates (each done in triplicate). (**D** and **E**) Control and *BEND3*-knockout OCI-AML2-Cas9 cells were treated with increasing concentrations of TAK-243 alone and in combination with 0.5 μM Ko143 (**D**) or 0.5 μM zosuquidar (**E**) for 72 hours. Cell growth and viability was measured by the MTS assay. Inset: the IC_50_ values (nM) are shown. Data points represent means ± SEM of 3–4 independent experiments.

Of the 49 known human ABC transporters, 12 have been reported to be commonly implicated in MDR (17, 18). To determine the most likely transporter for which TAK-243 might serve as a substrate, we correlated publicly available mRNA expression data of these 12 transporters and the IC_50_ of TAK-243 across 30 cancer cell lines for which TAK-243 sensitivity has been reported (Supplemental Table 3) (2). Breast cancer resistance protein (BCRP) displayed the strongest correlation between expression and TAK-243 sensitivity, with cells having the highest expression of BCRP being most resistant to the drug (*r* = 0.83; *P* < 0.0001). MDR-associated protein 2 (MRP2) also displayed a weaker but statistically significant correlation (*r* = 0.51; *P* < 0.0038). All the other transporters in our analysis did not correlate with sensitivity to TAK-243 (Figure 5, A and B, and Supplemental Figure 1).

These data suggest BCRP (encoded by *ABCG2*) and MRP2 may mediate TAK-243 efflux, and changes in BCRP and/or MRP2 expression may explain the resistance to TAK-243 after *BEND3* knockout. To test this hypothesis, we measured mRNA expression of *ABCG2*, *ABCC2* (encoding MRP2), as well as *ABCB1* (encoding P-glycoprotein, P-gp) in *BEND3*-knockout versus control OCI-AML2-Cas9 cells. As assessed by quantitative reverse transcription PCR (RT-qPCR), *BEND3* knockout increased *ABCG2* mRNA expression by 15-fold, while having no significant effect on *ABCC2* or *ABCB1* expression (Figure 5C). Thus, we decided to focus our investigation on BCRP. To test the functional importance of BCRP in explaining resistance to TAK-243 after *BEND3* knockout, we treated *BEND*3 knockout and control OCI-AML2-Cas9 cells with increasing concentrations of TAK-243 alone and in combination with either the selective BCRP inhibitor Ko143 (19, 20), or zosuquidar, a selective P-gp inhibitor (21). Inhibition of BCRP but not P-gp resensitized *BEND3*-knockout cells to TAK-243 (Figure 5, D and E).

**Figure 6:**
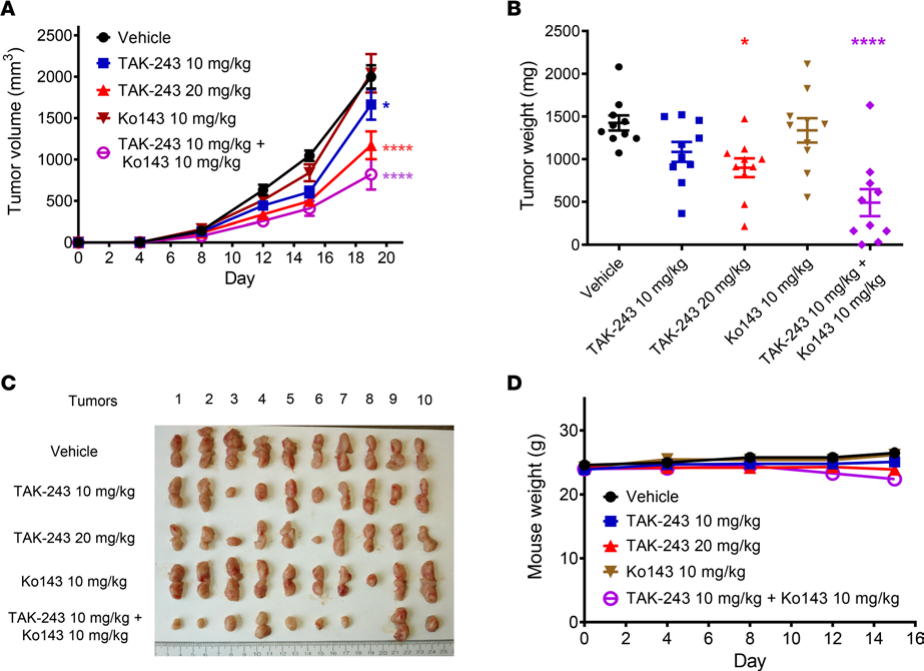
Chemical inhibition of BCRP sensitizes *BEND3*-knockout AML tumors to TAK-243 in vivo. (**A**) *BEND3*-knockout OCI-AML2 cells (1 × 10^6^) were injected subcutaneously into the flanks of SCID mice. When the tumors became palpable, mice were randomly divided into 5 groups (*n* = 10 per group) and treated with vehicle (10% HPBCD in water), TAK-243 (10 or 20 mg/kg), Ko143 10 mg/kg, or a combination of TAK-243 10 mg/kg + Ko143 10 mg/kg subcutaneously twice weekly for 3 weeks. Asterisks shown denote significantly different final tumor volumes in treated groups compared with vehicle, determined using repeated-measure 2-way ANOVA and Sidak’s multiple comparisons test. (**B**) After 3 weeks, mice were euthanized and tumors harvested and weighed. Significance of difference was determined using 1-way ANOVA and Tukey’s multiple comparisons test. (**C**) Images of tumors harvested from the 5 groups are shown. (**D**) Mice were weighed every 2–4 days. Data points (**A**, **B**, and **D**) represent means ± SEM. *P ≤ 0.05; ****P ≤ 0.0001.

To test the functional importance of BCRP in TAK-243 sensitivity in vivo, *BEND3*-knockout OCI-AML2 cells were injected subcutaneously into SCID mice. After the tumors became palpable, mice were treated with vehicle, TAK-243, Ko143 10 mg/kg, or a combination of Ko143 and TAK-243. Ko143 alone did not significantly affect tumor growth. However, systemic administration of the BCRP inhibitor sensitized tumors to TAK-243 without increased toxicity as evidenced by nonsignificant changes in body weight (Figure 6, A–D).

### BEND3 knockout confers partial cross-resistance to related adenosine sulfamates and selected MDR substrates.

**Figure 7:**
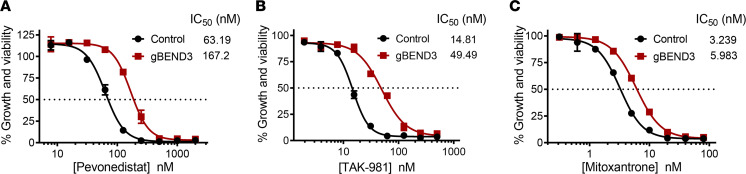
*BEND3* knockout confers partial cross-resistance to related adenosine sulfamates and selected MDR substrates. (**A**–**C**) Control and *BEND3*-knockout OCI-AML2-Cas9 cells were treated with increasing concentrations of pevonedistat (MLN4924) (**A**), TAK-981 (**B**), and mitoxantrone (**C**) for 72 hours. Cell growth and viability was measured by the MTS assay. Inset: the IC_50_ values (nM) are shown. Data points represent means ± SEM of 3 independent experiments.

To determine whether *BEND3* knockout confers resistance to other cytotoxic agents, we treated *BEND3*-knockout and control OCI-AML2-Cas9 cells with increasing concentrations of 6 related and unrelated drugs. The drugs evaluated were the NEDD8-activating enzyme (NAE) inhibitor pevonedistat (MLN4924/TAK-924), the SUMO-activating enzyme (SAE) inhibitor TAK-981, the proteasome inhibitor bortezomib, the endoplasmic reticulum stressors thapsigargin and tunicamycin, as well as the chemotherapeutic agent mitoxantrone, a well-known BCRP substrate (22–26). *BEND3* knockout conferred partial cross-resistance to pevonedistat, TAK-981, and mitoxantrone with a 2.6-, 3.3-, and 1.85-fold increase in their IC_50_ values (Figure 7, A–C). In contrast, knockout of *BEND3* displayed no cross-resistance to bortezomib, thapsigargin, or tunicamycin (Supplemental Figure 2).

### TAK-243 is a substrate for BCRP in cell lines of different origins.

**Figure 8:**
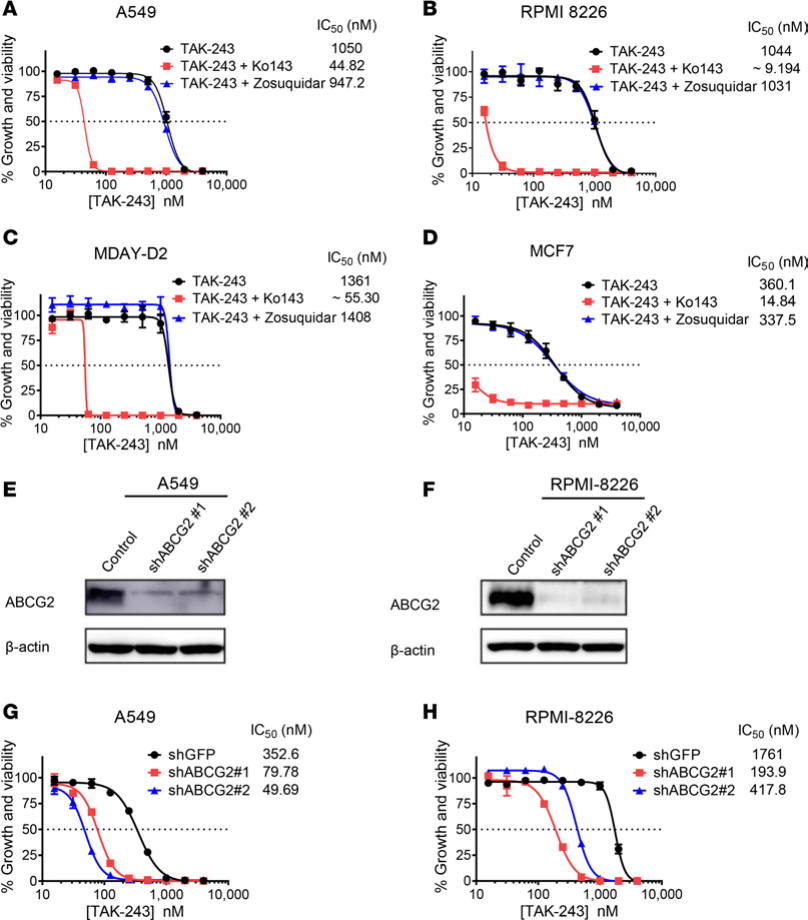
TAK-243 is a substrate for BCRP in cell lines of different origins. (**A**–**D**) A549 (**A**), RPMI 8226 (**B**), MDAY-D2 (**C**), and MCF7 (**D**) cells were treated with increasing concentrations of TAK-243 alone and in combination with either 0.5 μM Ko143 (BCRPi) or 0.5 μM zosuquidar (P-gpi) for 72 hours. Cell growth and viability was measured by the MTS assay. Inset: the IC_50_ values (nM) are shown. Data points represent means ± SEM. (**E** and **F**) A549 (**E**) and RPMI 8226 (**F**) cells were stably transduced with nontargeting or ABCG2-targeting shRNAs. After transduction, whole cell lysates were prepared, and levels of ABCG2 and β-actin serving as a loading control were measured by immunoblotting. (**G** and **H**) Control and ABCG2 knockdown cells of A549 (**G**) and RPMI 8226 (**H**) were treated with increasing concentrations of TAK-243 for 72 hours. Cell growth and viability was measured by the MTS assay. Inset: the IC_50_ values (nM) are shown. Data points represent means ± SEM of 3 independent experiments.

To determine whether BCRP mediates resistance to TAK-243 in other cell lines, we treated A549 lung cancer cells, MCF7 breast cancer cells, MDAY-D2 lymphosarcoma cells (27), and RPMI 8226 myeloma cells with TAK-243 alone and in combination with Ko143 or zosuquidar. Inhibition of BCRP with Ko143 sensitized all cell lines to TAK-243 with a potentiation up to 114-fold, while P-gp inhibition with zosuquidar had no impact on the response to TAK-243 (Figure 8, A–D).

To confirm these findings using a genetic approach, we knocked down *ABCG2* in A549 and RPMI 8226 cells using 2 distinct shRNAs and confirmed target knockdown by immunoblotting (Figure 8, E and F). Using the MTS assay, shRNA-mediated knockdown of *ABCG2* sensitized A549 and RPMI 8226 cells to TAK-243 and reduced the IC_50_ of the drug by 7- and 9-fold, respectively (Figure 8, G and H).

## Discussion

TAK-243 is a selective, mechanism-based UBA1 inhibitor with a broad preclinical efficacy in solid and hematologic malignancies and has entered phase I clinical trials (2, 10–13). In this study, we evaluated the regulators of sensitivity to TAK-243 in AML with potential implications in other malignancies using a genome-wide CRISPR/Cas9 knockout screen. From this screen, we identified *BEND3* as the top hit whose knockout conferred resistance to TAK-243.

BEND3 is a transcriptional repressor that interacts with chromatin-modifying complexes and induces repressive histone and DNA methylation changes resulting in transcriptional repression (28, 29). While *BEND3* knockout conferred resistance to TAK-243 in vitro and in vivo, it did not alter basal cell proliferation, consistent with publicly available data from pancancer RNA interference and CRISPR/Cas9 dropout screens showing *BEND3* is not an essential gene with no significant cell depletion upon knockdown or knockout (30).

Our study demonstrated that knockout of *BEND3* attenuated TAK-243 effects on poly- and mono-ubiquitylation of protein substrates and alleviated ER stress. Previous studies have shown that the induction of ER stress by TAK-243 is functionally important for TAK-243–induced cell death (2, 10–12).

Through subsequent experiments, we demonstrated that knockout of *BEND3* upregulates the MDR protein BCRP, resulting in increased efflux of the drug, reduced binding to UBA1, and consequently reduced UBA1 inhibition. The upregulation of MDR proteins leads to excessive efflux of structurally and mechanistically diverse drugs and is an important mechanism of drug resistance (31). BCRP has been reported to mediate the resistance of many unrelated anticancer drugs, including doxorubicin (23), etoposide (32), imatinib (33), methotrexate (34), and mitoxantrone (23, 35), among others (16, 17, 31). In keeping with this, our results showed the TAK-243–resistant *BEND3*-knockout cells were cross-resistant to the known BCRP substrate mitoxantrone. In AML, high expression of BCRP has been correlated to chemotherapy resistance, poor prognosis, and unfavorable therapeutic outcomes (36–40).

To our knowledge, no prior studies have implicated drug efflux pumps as mechanisms of resistance to TAK-243 or the related adenosine sulfamates, including pevonedistat and the SAE inhibitor ML-792 (41). Pevonedistat has been extensively studied in preclinical settings and in over 30 clinical trials; however, the upregulation of MDR proteins has not been reported as a mechanism of resistance to this drug. Instead, on-target missense mutations in *UBA3* (the gene encoding the active NAE subunit) have been reported to mediate acquired resistance to pevonedistat in preclinical systems (42–44). Analogous on-target missense mutations in *UBA1* have also been associated with TAK-243 resistance (10, 45). Here, we report for the first time to our knowledge that TAK-243 serves as a substrate for BCRP whose upregulation upon *BEND3* knockout confers resistance to the drug and potentially related adenosine sulfamates.

TAK-243 has been preclinically evaluated in several malignancies; however, the determinants of sensitivity remain largely unknown (2, 10–13). Hyer et al. investigated whether the sensitivity of TAK-243 was related to UBA1 expression levels or cell line proliferation rates as assessed by doubling time but found no significant correlation (2). In our study, we demonstrated that TAK-243 sensitivity strongly correlated with BCRP expression levels in cancer cell lines of different origins. We also found that selectively targeting BCRP with chemical inhibitors or shRNA-mediated knockdown sensitized cell lines intrinsically resistant to TAK-243 as a result of their high BCRP expression. Modulation of MDR proteins with inhibitors such as zosuquidar and tariquidar has been investigated in clinical trials as a strategy to sensitize certain malignancies to chemotherapy (46, 47). In such settings, it should be noted that while BCRP inhibitors may sensitize cancer cells to TAK-243, they may also lead to a narrower therapeutic window by exposing cells, normally protected from xenobiotics by high BCRP expression, to higher concentrations of the drug (48, 49). Therefore, this strategy may be used with caution in cases where toxicity can be managed or minimized.

Expression of BCRP and other MDR proteins is regulated by multiple transcriptional and posttranscriptional mechanisms as well as alterations in cellular signaling. In this respect, the promoter methylation status of *ABCG2* under basal conditions or in response to chemotherapy was reported to control BCRP expression levels in multiple myeloma cell lines and patient samples (50). MicroRNAs have also been implicated in regulating BCRP and other MDR proteins (33, 51–53). In addition, hormonal alterations have been reported to alter cell signaling and subsequently BCRP expression in breast cancer (54, 55). In our study, we demonstrated that BEND3 is important for regulating BCRP expression. Given its role as a transcriptional repressor, we speculate BEND3 regulates BCRP expression by inducing histone and DNA methylation changes at the promoter region of *ABCG2*. As per our CRISPR/Cas9 screen data, the histone methyltransferase *KMT5B* (*SUV420H1*) ranked as a second hit after *BEND3*. A related enzyme, KMT5C (SUV4-20H2), has been reported to interact with BEND3 in coimmunoprecipitation assays (28). Loss of BEND3 has also been reported to increase histone H3K4 trimethylation and DNA methylation of the ribosomal DNA promoter, silencing ribosomal DNA expression (29). Therefore, it is possible that BEND3 may interact with KMT5B to alter the methylation of *ABCG2* promoter, resulting in expression changes.

In summary, our study demonstrates TAK-243 is a substrate for the ABC efflux transporter BCRP. Moreover, TAK-243 sensitivity correlates with BCRP expression levels in cancer cell lines of different origins, suggesting BCRP expression can serve as a predictive biomarker of TAK-243 response and a potential therapeutic target for synergistic combinations with TAK-243. Our study also reports, for the first time to our knowledge, BEND3 as a transcriptional regulator of BCRP expression and that lack of BEND3 expression confers resistance to TAK-243 and potentially other BCRP substrates.

## Methods

### Chemical and reagents.

TAK-243 (formerly known as MLN7243) was provided by Millennium Pharmaceuticals, Inc., a wholly owned subsidiary of Takeda Pharmaceutical Company Limited, and purchased from Active Biochem (catalog A-1384), and pevonedistat (MLN4924/TAK-924) was provided by Dalton Medicinal Chemistry. Bortezomib (catalog S1013) was purchased from Selleckchem, TAK-981 from ChemiTek (catalog CT-TAK981), mitoxantrone (catalog M6545) and HPBCD (catalog H107) from MilliporeSigma, and tunicamycin (catalog 3516), thapsigargin (catalog 1138), zosuquidar (catalog 5456), and Ko143 (catalog 3241) from Tocris. Ko143 for animal experiments was purchased from MedChemExpress (catalog HY-10010).

### Cell culture.

OCI-AML2, K562, MV4-11, and RPMI 8226 cells were cultured in IMDM; NB4, U937, MDAY-D2, and Jurkat cells in RPMI medium; and A549, Hep G2, and MCF7 cells in DMEM. All culture media were supplemented with 10% fetal bovine serum and appropriate antibiotics. All cells were incubated at 37°C, 5% CO_2_ with 95% humidity. All cell lines were obtained from the American Type Culture Collection (ATCC) except for OCI-AML2 and MDAY-D2 cells that were obtained as a gift from Mark Minden (University Health Network, Toronto, Canada).

### Positive-selection genome-wide CRISPR/Cas9 knockout screen.

The genome-wide CRISPR/Cas9 knockout screen was performed in OCI-AML2 cells stably expressing Cas9 (OCI-AML2-Cas9). To generate these cells, OCI-AML2 cells were transduced with a Cas9 in a lentiviral vector, Lenti-Cas9-2A-Blast (Addgene plasmid 73310) (14). The cells were then selected with blasticidin (10 μg/mL) for 6 days. Single-cell clones were obtained by plating in a 96-well plate at a density of 0.4 cell/well, and a clonal population with high Cas9 expression was selected. OCI-AML2-Cas9 cells were transduced with a pooled library (90k library) of 91,320 gRNAs in lentiviral vectors targeting 17,232 genes at a ratio of 6 gRNAs per gene (14). These cells were transduced at a multiplicity of infection (MOI) of approximately 0.3–0.4 to obtain coverage of at least 200-fold per gRNA. One day posttransduction, cells were treated with puromycin (2 μg/mL) for 48 hours to select transduced cells. Cells were then treated with DMSO or TAK-243 at its IC_90_ (25 nM) or IC_99_ (30 nM) for 32 days. Genomic DNA was then extracted from cell populations; gRNA sequences were amplified by PCR and sequenced on an Illumina Hiseq2500. Data were analyzed using MAGeCK method (15).

### CRISPR/Cas9 knockout and shRNA-mediated knockdown experiments.

For CRISPR/Cas9 knockout experiments, OCI-AML2-Cas9 cells (5 × 10^6^) were resuspended in 5 mL of fresh media containing protamine sulfate (5 μg/mL). Viral supernatants (2 mL) of 2 distinct *BEND3*-targeting gRNAs encoded in pLCKO lentiviral vectors (gBEND3 #1 and #2) were added to cells to achieve an MOI of 0.3 (Addgene plasmid 73311; ref. 14). After 24 hours of incubation, cells were centrifuged at 600*g* for 5 minutes at 25°C and resuspended in fresh media containing puromycin (1.5 μg/mL). After 3 days of selection, cell lysates were collected, and knockout was then confirmed by immunoblotting. *BEND3* was also knocked out using a single-plasmid system encoding additional gRNAs. To do so, OCI-AML2 cells were transduced with lentiCRISPR v2 vectors encoding Cas9 and 3 distinct *BEND3*-targeting gRNAs (crV2-BEND3 #1-3) as described above (Addgene plasmid 52961; ref. 56). For shRNA-mediated knockdown experiments, *ABCG2*-targeting shRNAs were obtained from MilliporeSigma (product SHCLNG-NM_004827) and transduced into A549 and RPMI 8226 cells as described above. Sequences of *BEND3*-targeting gRNAs and *ABCG2*-targeting shRNAs are listed in Supplemental Table 4.

### Cytotoxicity assays.

CellTiter 96 AQueous MTS Reagent Powder was purchased from Promega (catalog G1111) and annexin V-FITC apoptosis kit from Biovision (catalog K101-400). The MTS and annexin V/PI assays were performed as per the manufacturer’s instructions. For the MTS assay, the cells were counted and seeded in 96-well plates at the following densities: OCI-AML2 (25,000/well), K562 (10,000/well), MV4-11 (25,000/well), RPMI 8226 (25,000/well), NB4 (25,000/well), U937 (10,000/well), MDAY-D2 (10,000/well), and Jurkat (10,000/well) and treated with increasing concentrations of the drug(s) under investigation. After 72 hours of incubation, the MTS solution was directly added to the media at a ratio of 1:5, and absorbance was measured at 490 nm using SpectraMax Microplate Reader (Molecular Devices). The growth and viability were then calculated as a percentage of the untreated cells, and concentration-response curves were constructed and IC_50_ calculated using the nonlinear regression function in GraphPad Prism (Version 6.03, GraphPad Software Inc.). For the annexin V/PI assay, OCI-AML2 cells were seeded in a 24-well plate at a plating density of 1 × 10^5^ cells/mL and treated with increasing concentrations of TAK-243. After 96 hours of incubation, media were collected, and cells were washed with phosphate-buffered saline (PBS), centrifuged at 900*g* at 25°C for 10 minutes, and then resuspended in the binding buffer containing annexin V-FITC and PI. Unstained and single-stained cells were used as compensation controls. Flow cytometric analysis of samples was performed using BD FACSCanto flow cytometer (BD Biosciences).

For the colony-forming assay, control and *BEND3*-knockout OCI-AML2 cells were seeded in MethoCult H4100 medium (StemCell Technologies) in 35 mm gridded dishes at a plating density of 400 cells/dish (DMSO treated) or 1000 cells/dish (TAK-243 treated) and were incubated for 7 days to allow colonies to form. After incubation, colonies of at least 50 cells were counted, and plating efficiency (PE) was calculated from DMSO-treated controls using the following equation: #colonies counted/#cells seeded. The percentage viability of TAK-243–treated cells was then calculated using this equation: (#colonies counted/[#cells seeded × PE] × 100) as previously described (57). For the proliferation assays, DMSO- and TAK-243–treated OCI-AML2 cells were seeded at a density of 10^4^ cells/mL, and viable trypan blue–negative cells were counted every 2–3 days using a hemocytometer.

### Cellular thermal shift assay.

We conducted CETSA as previously described (58). In brief, cells were treated with increasing concentrations of TAK-243 for 1 hour. Cells were then washed with PBS and resuspended in PBS containing protease inhibitor cocktail (Thermo Fisher Scientific). Cells were heated at 54°C for 3 minutes in a thermal cycler (SimpliAmp, Applied Biosystems, Thermo Fisher Scientific). This temperature corresponds to the maximal thermal shift of UBA1 experimentally derived as previously described (10). Cell lysates were prepared by 4 freeze-thaw cycles in liquid nitrogen and a thermal cycler set at 25°C, respectively, with vigorous vortexing in between. Lysates were then centrifuged at 20,000*g* for 20 minutes, and supernatants were collected and frozen at –70°C until immunoblotting.

### Quantitative reverse transcription polymerase chain reaction.

Total RNA was isolated using the RNeasy Plus Mini Kit (QIAGEN) and reverse transcribed into cDNAs using SuperScript IV Reverse Transcriptase (Thermo Fisher Scientific). Equal cDNA amounts were then added to a PCR master mix (*Power* SYBR Green PCR Master Mix; Applied Biosystems, Thermo Fisher Scientific). RT-qPCR reactions were conducted using an ABI Prism 7900 sequence detection system (Applied Biosystems, Thermo Fisher Scientific). The relative gene expression was calculated by the 2^–ΔΔ^Ct method using 18s rRNA as a control. Primer sequences used in the study are listed in Supplemental Table 5.

### Immunoblotting.

To prepare whole cell lysates, cells were washed with PBS (pH = 7.4) and lysed with RIPA buffer followed by sonication and centrifugation at 15,000*g* for 20 minutes at 4°C. Supernatants were collected and total protein was quantified using the Bradford assay (Bio-Rad). Samples were then denatured by boiling at 95°C for 5 minutes. For CETSA lysates, samples were not sonicated and were heated at 70°C for 10 minutes. Proteins were loaded in equal amounts and then fractionated by 10% gels (unless otherwise specified) using SDS-PAGE. Proteins were transferred to PVDF membranes and then probed using appropriate primary and secondary antibodies (Supplemental Table 6).

### Determination of intracellular ATP levels.

Intracellular ATP levels were measured using a highly sensitive ATP Bioluminescence Assay Kit HS II (MilliporeSigma; catalog 11-699-709-001) as per the manufacturer’s guidelines. In brief, control and *BEND3*-knockout OCI-AML2 cells were washed with PBS and resuspended in the manufacturer’s dilution buffer, then seeded in triplicate in white 96-well microtiter plates at a plating density of 25,000 cells and a volume of 25 μL per well. Cells were then lysed by adding an equal volume of cell lysis buffer and incubating for 5 minutes at room temperature. A 50 μL of the luciferase reagent was then dispensed by automated injection, and luminescence was measured after a 1 s delay and integration for 1 s using Hidex Sense Microplate Reader (Hidex Inc.). Relative ATP levels in *BEND3*-knockout OCI-AML2 cells were calculated by normalizing the luminescence intensities obtained from the assay to control OCI-AML2 cells.

### Measurement of intracellular TAK-243 concentrations.

To assess TAK-243 concentrations in the cells, *BEND3*-knockout and control OCI-AML2 cells were seeded in triplicate in a 12-well plate at a density of 10 × 10^6^/well and then treated with increasing concentrations of the drug. After 1 hour of incubation, cells were collected and centrifuged at 800*g* at 4°C for 5 minutes, and media were removed by aspiration. The cells were then washed twice with drug-free PBS and kept on ice during processing. Cell pellets were then extracted with 50 μL of ice-cold acetonitrile containing internal standard. Cell extracts were centrifuged at 17,500*g* at 4°C for 10 minutes, followed by careful collection of 40 μL of the supernatant in HPLC vials, and were stored at –20°C until LC-MS analysis. To measure TAK-243 by LC-MS, we used an Acquity UPLC BEH C18 (2.1 × 50 mm, 1.7 μm) column using Acquity UPLC I-Class system. The mobile phase was 0.1% formic acid in water (solvent A) and 0.1% formic acid in acetonitrile (solvent B). A gradient starting at 95% solvent A going to 5% in 4.5 minutes, holding for 0.5 minutes, going back to 95% in 0.5 minutes, and equilibrating the column for 1 minute was employed. A Waters Synapt G2S QTof mass spectrometer equipped with an electrospray ionization source was used for mass spectrometric analysis.

### Animal studies.

To assess effect of *BEND3* knockout on TAK-243 response in vivo, control and *BEND3*-knockout OCI-AML2 cells (1 × 10^6^ trypan blue–negative viable cells) were injected subcutaneously (s.c.) into the right and left flanks of male SCID mice (Ontario Cancer Institute, Toronto, Canada), respectively. After the tumors became palpable, mice were randomly divided into 4 groups (*n* = 5 per group) and treated with vehicle (10% HPBCD in water) or TAK-243 at doses of 10, 15, and 20 mg/kg s.c. BIW for 3 weeks. Mice were weighed and tumor volumes were measured by caliper measurements every 2–3 days using the following equation: tumor volume in mm^3^ = tumor length in mm × width^2^ in mm × 0.5236 as previously described (59). At the end of the experiment, mice were euthanized and tumors excised for weighing.

To assess the impact of Ko143 on TAK-243 response in vivo, *BEND3*-knockout OCI-AML2 cells were similarly injected as described above. After the tumors became palpable, mice were randomly divided into 5 groups (*n* = 10 per group) and treated BIW with vehicle, TAK-243 at doses of 10 and 20 mg/kg s.c., Ko143 (dissolved in 10% DMSO/10% cremophor in 0.9% NaCl) at a dose of 10 mg/kg intraperitoneally, or a combination of TAK-243 10 mg/kg + Ko143 10 mg/kg where mice were injected with Ko143 2 hours before TAK-243. The selected dose of Ko143 was the maximally tolerated dose that could be given in combination with TAK-243.

### Data sets.

The CRISPR/Cas9 data sets have been deposited in the National Center for Biotechnology Information’s Gene Expression Omnibus database with accession number GSE164639 and can be accessed online at https://www.ncbi.nlm.nih.gov/geo/query/acc.cgi?acc=GSE164639

### Statistics.

GO enrichment analysis was performed for the significantly enriched genes (positive-selection FDR < 0.05) of the IC_90_ concentration arm using GSEA tool (60). Gene sets (each corresponding to a specific GO term) were considered significant if enriched with an FDR of less than 0.05. IC_50_ and IC_90_ values were calculated using the nonlinear regression function in GraphPad Prism software version 6.03 (GraphPad Software Inc.). To analyze flow cytometry data, BD FACSDiva Software 6.0 (BD Biosciences) and FlowJo version 7.7.1 (FlowJo, LLC) were used.

GraphPad Prism software was used to perform all statistical analyses. To calculate the significance of differences between means, unpaired *t* test (2 groups), 1-way ANOVA and appropriate multiple comparisons test (˃2 groups), and 2-way ANOVA (2 independent variables) and appropriate multiple comparisons test were used. All experiments were performed in triplicate with at least 3 biological replicates unless otherwise specified.

### Study approval.

All animal studies were carried out according to the regulations of the Canadian Council on Animal Care and with the approval of the local ethics review board at University Health Network.

## Author contributions

SHB, AA, KN, ZB, KA, GET, NM, RH, T Ketela, MS, MA, T Kiyota, and RA conducted experiments and analyzed data; ADS supervised research, data analysis, and interpretation; SHB and ADS conceived the project and designed the experiments; and SHB and ADS wrote the manuscript. All authors reviewed and edited the manuscript.

## Supplementary Material

Supplemental data

Supplemental Table 3

Supplemental Table 4

## Version 1 01/21/2021

In-Press Preview

## Version 2 03/08/2021

Electronic publication
