# Supplementary material for: A genome-wide CRISPR/Cas9 screen in acute myeloid leukemia cells identifies regulators of TAK-243 sensitivity
Source: JCI Insight. 2021 Mar 8;6(5):e141518. doi: 10.1172/jci.insight.141518 (PMC8021101; doi:10.1172/jci.insight.141518)
Supplement: Supplemental data [file jciinsight-6-141518-s053.pdf]

A genome-wide CRISPR/Cas9 screen in acute myeloid leukemia cells identifies regulators of TAK-243 sensitivity

Samir H. Barghout[1,2,3], Ahmed Aman[4,5], Kazem Nouri[1], Zachary Blatman[1,6], Karen Arevalo[1,6], Geethu Thomas[1], Neil MacLean[1], Rose Hurren[1], Troy Ketela[1], Mehakpreet Saini[4], Moustafa Abohawya[7], Taira Kiyota[4], Rima Al-Awar[4,8], and Aaron D. Schimmer[1,2,6*]

[1]Princess Margaret Cancer Centre, University Health Network, Toronto, ON, Canada; [2]Department of Medical Biophysics, Faculty of Medicine, University of Toronto, Toronto, ON, Canada;

[3]Department of Pharmacology & Toxicology, Faculty of Pharmacy, Tanta University, Tanta; Egypt;

[4]Drug Discovery Program, Ontario Institute for Cancer Research, Toronto, ON, Canada;

[5]Leslie Dan Faculty of Pharmacy, University of Toronto, Toronto, ON, Canada;

[6]Institute of Medical Science, Faculty of Medicine, University of Toronto, Toronto, ON, Canada;

[7]Department of Biomedical Sciences, Zewail City of Science and Technology, Giza, Egypt;

[8]Department of Pharmacology & Toxicology, University of Toronto, Toronto, ON, Canada.

* Corresponding author:
Dr. Aaron D. Schimmer
Princess Margaret Cancer Research Tower, Room 8-706
101 College St, Toronto, ON, M5G 1L7, Canada.
Tel: 416-946-2838
E-mail: aaron.schimmer@uhn.ca

Running title: Regulators of TAK-243 sensitivity

Key words: TAK-243; UBA1; Ubiquitin; AML; BEND3; ABCG2; ABC transporters; Anticancer drug sensitivity

Table S1 Top hits in the IC_{90} arm of the CRISPR/Cas9 knockout screen as assessed by the MAGeCK algorithm

Rank	Gene	Number	Score	*p*-value	FDR	Good gRNA
**1**	BEND3	6	6.55E-21	2.87E-07	0.001238	6
**2**	FLCN	6	7.30E-13	2.87E-07	0.001238	6
**3**	SUV420H1	6	3.83E-11	2.87E-07	0.001238	5
**4**	DAZAP1	6	7.16E-11	2.87E-07	0.001238	6
**5**	FBXL20	6	2.19E-08	8.62E-07	0.002475	6
**6**	EPC2	6	2.87E-08	8.62E-07	0.002475	4
**7**	CDK2	6	6.33E-08	1.44E-06	0.003536	6
**8**	ZNF800	5	2.48E-07	2.58E-06	0.005569	4
**9**	BAHD1	6	7.91E-07	5.46E-06	0.010451	6
**10**	RC3H1	6	1.55E-06	1.12E-05	0.014851	5
**11**	RRAGA	6	1.64E-06	1.12E-05	0.014851	6
**12**	ZNF787	6	1.67E-06	1.12E-05	0.014851	6
**13**	HDAC1	6	5.85E-06	3.30E-05	0.040594	6
**14**	LINS	6	6.18E-06	3.53E-05	0.040594	6
**15**	FOXP3	6	1.03E-05	5.00E-05	0.053837	4
**16**	SFMBT2	6	1.18E-05	5.95E-05	0.058581	5
**17**	ACSL4	6	1.25E-05	6.12E-05	0.058581	6
**18**	QKI	6	2.70E-05	0.000123	0.109586	5
**19**	ZFR	6	2.81E-05	0.00013	0.109586	5
**20**	TFPI2	6	2.95E-05	0.000136	0.109586	6
**21**	CCDC43	6	3.00E-05	0.00014	0.109586	6
**22**	DROSHA	6	3.95E-05	0.000193	0.144641	3
**23**	UBE2F	3	5.17E-05	0.000254	0.18255	3
**24**	SRM	6	5.65E-05	0.000277	0.187069	4
**25**	MAT2B	6	5.86E-05	0.000285	0.187069	6
**26**	SMAD7	6	6.22E-05	0.000296	0.187069	6
**27**	SLC30A10	6	6.88E-05	0.000333	0.187069	4
**28**	CALHM3	6	7.09E-05	0.000339	0.187069	4
**29**	TIAL1	6	7.15E-05	0.000341	0.187069	5
**30**	SAFB2	6	7.33E-05	0.000346	0.187069	5
**31**	AZIN1	3	7.57E-05	0.000357	0.187069	3
**32**	HEXB	6	7.59E-05	0.000358	0.187069	3
**33**	ZC3HAV1	6	8.32E-05	0.000386	0.195836	6

Good guide RNAs (gRNAs): gRNAs whose ranking is below the alpha cutoff; Score: Robust Ranking Aggregation (RRA) algorithm score of enriched/depleted gRNAs; FDR: False discovery rate

Table S2 Top hits in the IC_{99} arm of the CRISPR/Cas9 knockout screen as assessed by the MAGeCK algorithm

Rank	Gene	Number	Score	*p*-value	FDR	Good gRNA
**1**	BEND3	6	1.03E-19	2.87E-07	0.001238	6
**2**	SUV420H1	6	4.47E-12	2.87E-07	0.001238	4
**3**	DAZAP1	6	1.23E-10	2.87E-07	0.001238	5
**4**	CDK2	6	2.13E-09	2.87E-07	0.001238	5
**5**	TIAL1	6	3.58E-07	1.44E-06	0.00495	4
**6**	ACSL4	6	1.18E-06	3.73E-06	0.010726	4
**7**	OPRK1	6	9.33E-06	2.50E-05	0.061528	3
**8**	RRAGA	6	2.27E-05	6.17E-05	0.133045	3
**9**	FLCN	6	2.79E-05	7.50E-05	0.143564	3
**10**	EIF4G1	6	3.98E-05	0.000101	0.174752	3
**11**	SAFB2	6	5.01E-05	0.000124	0.193969	3

Table S3 TAK-243 sensitivity data

#	Cell Line	TAK-243 IC_{50} (nM)*
1	NCIH82_LUNG	6
2	K562_HAEMATOPOIETIC_AND_LYMPHOID_TISSUE	9
3	NB4_HAEMATOPOIETIC_AND_LYMPHOID_TISSUE	10
4	JURKAT_HAEMATOPOIETIC_AND_LYMPHOID_TISSUE	10
5	NCIH69_LUNG	11
6	MM1S_HAEMATOPOIETIC_AND_LYMPHOID_TISSUE	12
7	NCIH520_LUNG	13
8	MV411_HAEMATOPOIETIC_AND_LYMPHOID_TISSUE	15
9	WSUDLCL2_HAEMATOPOIETIC_AND_LYMPHOID_TISSUE	16
10	HL60_HAEMATOPOIETIC_AND_LYMPHOID_TISSUE	17
11	U937_HAEMATOPOIETIC_AND_LYMPHOID_TISSUE	17
12	THP1_HAEMATOPOIETIC_AND_LYMPHOID_TISSUE	19
13	HCT116_LARGE_INTESTINE	20
14	OCIAML2_HAEMATOPOIETIC_AND_LYMPHOID_TISSUE	27
15	SW480_LARGE_INTESTINE	44
16	U266B1_HAEMATOPOIETIC_AND_LYMPHOID_TISSUE	44
17	MIAPACA2_PANCREAS	45
18	SW48_LARGE_INTESTINE	56
19	HEPG2_LIVER	60
20	NCIH929_HAEMATOPOIETIC_AND_LYMPHOID_TISSUE	109
21	HCC1954_BREAST	123
22	MDAMB231_BREAST	172
23	CALU6_LUNG	174
24	HT29_LARGE_INTESTINE	308
25	MCF7_BREAST	360
26	A375_SKIN	381
27	LOVO_LARGE_INTESTINE	450
28	A549_LUNG	1187
29	NCIH460_LUNG	1310
30	RPMI8226_HAEMATOPOIETIC_AND_LYMPHOID_TISSUE	1366

Sensitivity data were obtained from Ref. (1), Ref. (2) and experimental data from our laboratory

Table S4 Sequences of *BEND3*-targeting gRNAs and *ABCG2* mRNA-targeting shRNAs

ID	Sequence (5'→3')
***BEND3*-targeting gRNAs**	
**gBEND3 #1**	TCAGGAGCCGCAGTGACGAG
**gBEND3 #2**	GGATGAGCTTGATGCGGGAG
**crV2 BEND3 #1**	GGATGAGCTTGATGCGGGAG
**crV2 BEND3 #2**	TGAACAGTACAGCTGCTACG
**crV2 BEND3 #3**	AGTAGACCTCCACATAGTTG
***ABCG2*-targeting shRNAs**	
**shABCG2 #1**	CCTGCCAATTTCAAATGTAAT
**shABCG2 #2**	TAACATCTGCTATCGAGTAAA

Table S5 Primers used for RT-qPCR

ID	F/R	Sequence (5'→3')
ABCG2	F	GTGGCCTTGGCTTGTATGAT
**(BCRP)**	R	GATGGCAAGGGAACAGAAAA
ABCB1	F	GCTCCTGACTATGCCAAAGC
**(P-gp)**	R	TCTTCACCTCCAGGCTCAGT
ABCC2	F	TGCTTCCTGGGGATAATCAG
**(MRP2)**	R	CACGGATAACTGGCAAACCT

Table S6 Antibodies used in Western blotting, their sources and dilution

Primary antibody	Source	Clone	Catalog#	Dilution
ABCG2/BCRP	Cell Signaling	D5V2K	42078S	Primary: 1:1000<br>Secondary: 1:1000
ATF4	Santa Cruz	C-20	sc-200	Primary: 1:100<br>Secondary: 1:1000
BEND3	Proteintech	-	23101-1-AP	Primary: 1:500<br>Secondary: 1:1000
Cas9	Cell Signaling	7A9-3A3	14697	Primary: 1:1000<br>Secondary: 1:1000
CHOP	Sigma	-	G6916	Primary: 1:500<br>Secondary: 1:1000
GAPDH	Cell Signaling	14C10	2118	Primary: 1:4000<br>Secondary: 1:2000
PARP	Cell Signaling	-	9542	Primary: 1:1000<br>Secondary: 1:1000
p-JNK$^{Thr183/Tyr185}$	Cell Signaling	-	9251	Primary: 1:1000<br>Secondary: 1:1000
UBA1	Santa Cruz	2G2	sc-53555	Primary: 1:300<br>Secondary: 1:1000
UBA2	Santa Cruz	28	sc-136359	Primary: 1:500<br>Secondary: 1:1000
UBA3	Santa Cruz	E-5	sc-377272	Primary: 1:400<br>Secondary: 1:1000
UBA6	Cell Signaling	-	13386	Primary: 1:1000<br>Secondary: 1:1000
Ubiquitin	Cell Signaling	-	3933	Primary: 1: 500<br>Secondary: 1:1000
Ubiquityl-Histone H2A (Lys119)	Cell Signaling	D27C4	8240	Primary: 1:1000<br>Secondary: 1:1000
β-actin	Santa Cruz	AC-15	sc-69879	Primary: 1:10,000<br>Secondary: 1:5,000
γH2AX^{Ser139}	EMD Millipore	JBW301	05-636	Primary: 1:600<br>Secondary: 1:1000

Supplementary References

1. Hyer ML, Milhollen MA, Ciavarri J, Fleming P, Traore T, Sappal D, et al. A small-molecule inhibitor of the ubiquitin activating enzyme for cancer treatment. *Nature medicine.* 2018;24(2):186-93.
2. Zhuang J, Shirazi F, Singh RK, Kuiatse I, Wang H, Lee HC, et al. Ubiquitin-activating enzyme inhibition induces an unfolded protein response and overcomes drug resistance in myeloma. *Blood.* 2019.

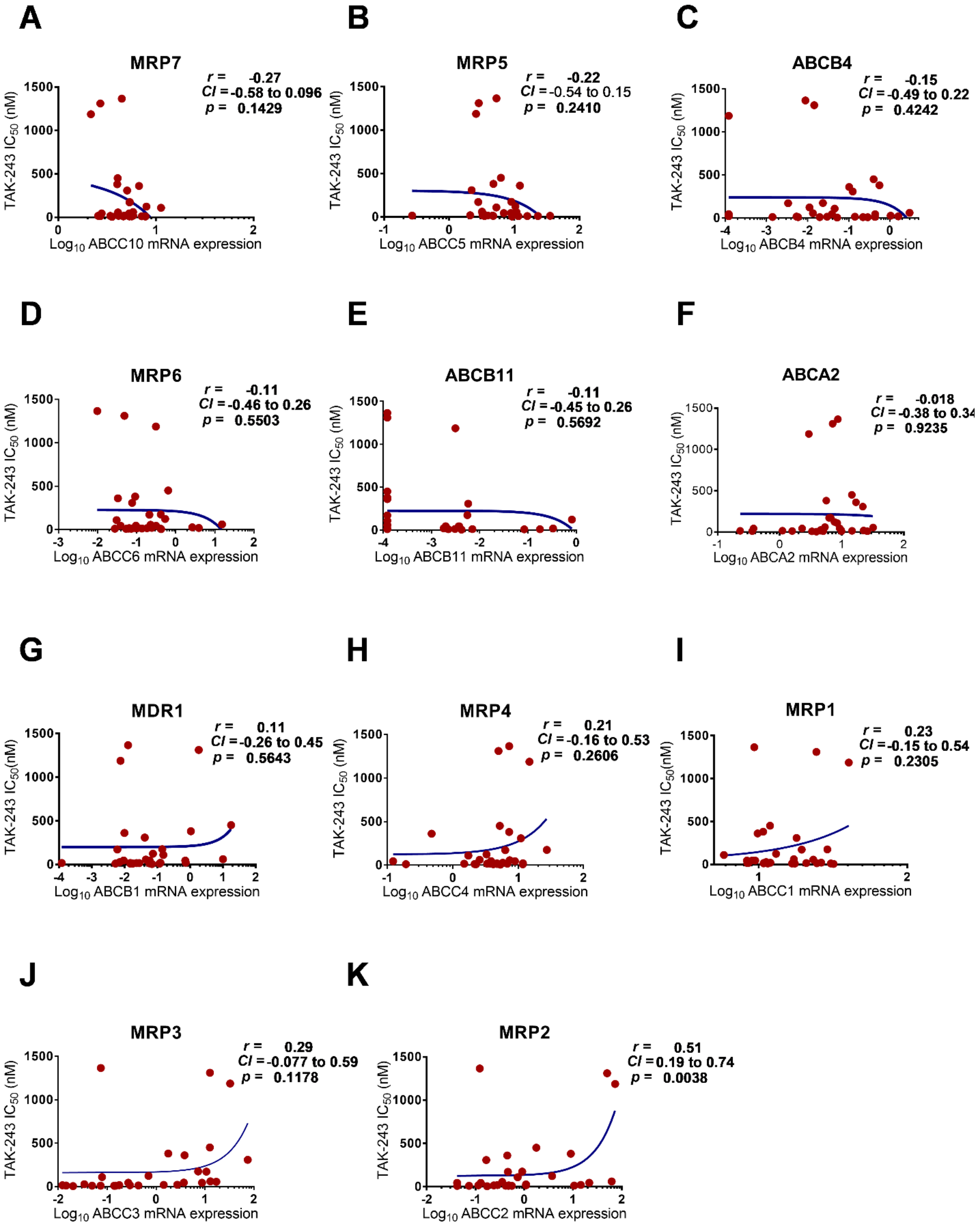

Figure S1 Correlation of ABC transporter expression and TAK-243 sensitivity. A-K) Correlation curves of the mRNA expression of ABC transporters commonly involved in drug multidrug resistance and TAK-243 sensitivity (as measured by IC_{50}). Data points represent the 30 cell lines used in the analysis. A logarithmic scale was used for the X-axis to display all the data points over a wide range. Insert: the Pearson correlation coefficient (r), confidence interval (CI) and significance of correlation (as assessed by *p* value). Transporter names are shown on the graphs.

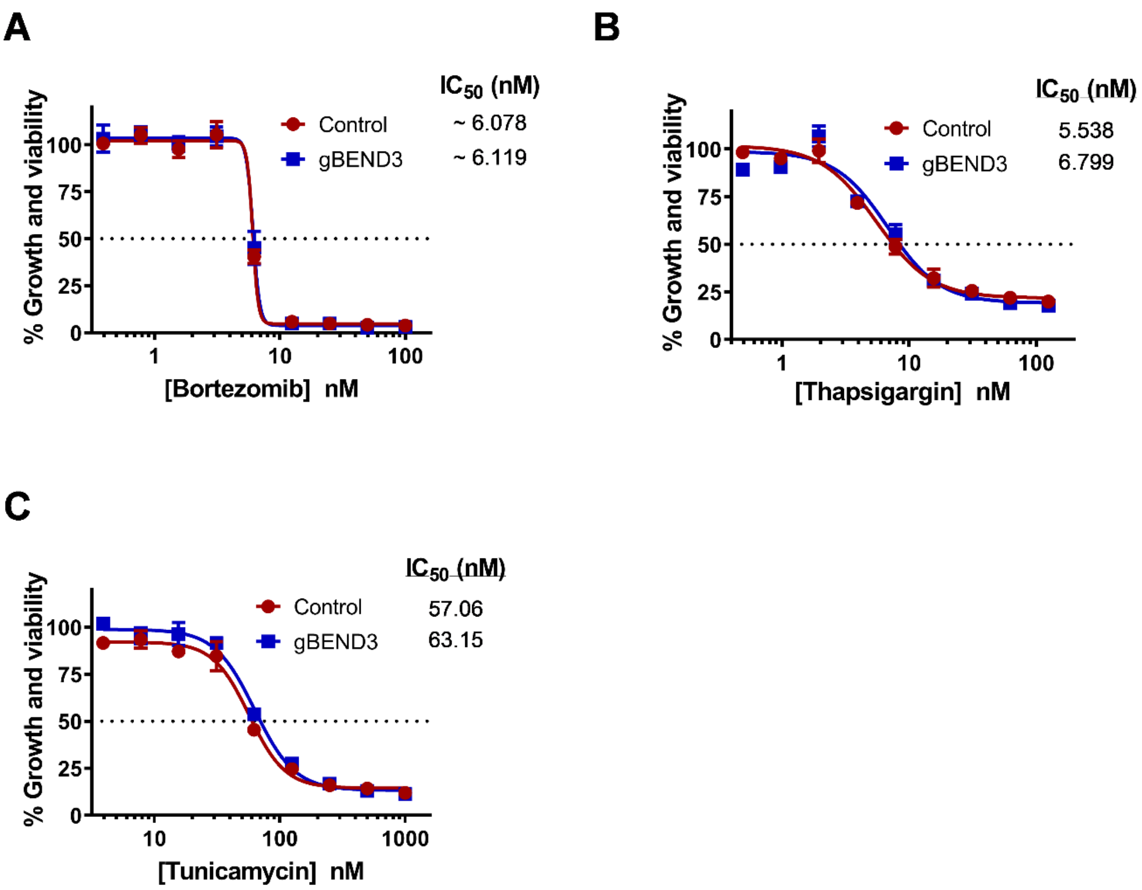

Figure S2 *BEND3* knockout does not affect response to proteasome inhibitors or ER stressors. A-C) Control and *BEND3* knockout OCI-AML2-Cas9 cells were treated with increasing concentrations of bortezomib (A), thapsigargin (B), and tunicamycin (C) for 72 h. Cell growth and viability was measured by the MTS assay. Insert: the IC_{50} values (nM) are shown. Data points represent means ± SEM of at least 3 independent experiments.
